# Paralytic Shellfish Toxins and Cyanotoxins in the Mediterranean: New Data from Sardinia and Sicily (Italy)

**DOI:** 10.3390/microorganisms5040072

**Published:** 2017-11-16

**Authors:** Antonella Lugliè, Maria Grazia Giacobbe, Elena Riccardi, Milena Bruno, Silvia Pigozzi, Maria Antonietta Mariani, Cecilia Teodora Satta, Daniela Stacca, Anna Maria Bazzoni, Tiziana Caddeo, Pasqualina Farina, Bachisio Mario Padedda, Silvia Pulina, Nicola Sechi, Anna Milandri

**Affiliations:** 1 Dipartimento di Architettura, Design e Urbanistica, University of Sassari, Via Piandanna 4, 07100 Sassari, Italy; luglie@uniss.it (A.L.); marianim@uniss.it (M.A.M.); dstacca@uniss.it (D.S.); tcaddeo@uniss.it (T.C.); pasqui81@hotmail.it (P.F.); bmpadedda@uniss.it (B.M.P.); pulinasi@uniss.it (S.P.); sechi@uniss.it (N.S.); 2 Istituto per l’Ambiente Marino Costiero, CNR, Spianata S. Raineri 86, 98122 Messina, Italy; mariagrazia.giacobbe@iamc.cnr.it; 3 Fondazione Centro Ricerche Marine, National Reference Laboratory for Marine Biotoxins, Viale A. Vespucci 2, 47042 Cesenatico (FC), Italy; elena.riccardi@centroricerchemarine.it (E.R.); silvia.pigozzi@centroricerchemarine.it (S.P.); anna.milandri@centroricerchemarine.it (A.M.); 4 Environmental Quality and Fish Farming, Environment and Primary Prevention, Istituto Superiore di Sanità, V.le Regina Elena 299, 00161 Rome, Italy; milena.bruno@iss.it; 5 Agenzia Regionale per la Ricerca in Agricoltura (AGRIS), Servizio Ittico, S.S. Sassari-Fertilia Km 18,600, Bonassai, 07040 Olmedo, Italy; 6 Dipartimento di Ispezione degli Alimenti, Istituto Zooprofilattico Sperimentale della Sardegna G. Pegreffi, Via Duca degli Abruzzi 8, 07100 Sassari, Italy; bazzoni.annamaria@tiscali.it; 7 Department of Life and Environmental Sciences, University of Cagliari, Via Fiorelli 1, 09126 Cagliari, Italy

**Keywords:** paralytic shellfish toxins, microcystins, BMAA, *Alexandrium*, artificial lakes, Mediterranean

## Abstract

Harmful algal blooms represent a severe issue worldwide. They affect ecosystem functions and related services and goods, with consequences on human health and socio-economic activities. This study reports new data on paralytic shellfish toxins (PSTs) from Sardinia and Sicily (Italy), the largest Mediterranean islands where toxic events, mainly caused by *Alexandrium* species (Dinophyceae), have been ascertained in mussel farms since the 2000s. The toxicity of the *A. minutum*, *A. tamarense* and *A. pacificum* strains, established from the isolation of vegetative cells and resting cysts, was determined by high performance liquid chromatography (HPLC). The analyses indicated the highest toxicity for *A. pacificum* strains (total PSTs up to 17.811 fmol cell^−1^). The PSTs were also assessed in a strain of *A. tamarense*. The results encourage further investigation to increase the knowledge of toxic species still debated in the Mediterranean. This study also reports new data on microcystins (MCs) and β-*N*-methylamino-l-alanine (BMAA) from a Sardinian artificial lake (Lake Bidighinzu). The presence of MCs and BMAA was assessed in natural samples and in cell cultures by enzyme-linked immunosorbent assay (ELISA). BMAA positives were found in all the analysed samples with a maximum of 17.84 µg L^−1^. The obtained results added further information on cyanotoxins in Mediterranean reservoirs, particularly BMAA, which have not yet been thoroughly investigated.

## 1. Introduction

Harmful algal blooms (HABs) are recognized as one of the major threats in aquatic ecosystems worldwide [1,2,3,4]. HABs affect ecosystem functioning (e.g., biodiversity, energy flow and food chains) and, consequently, they negatively impact functions and related goods and services (e.g., food production, recreational and cultural activities), leading to economic consequences [5,6,7].

A major negative aspect of HABs is the production of toxic compounds which impact on human health [8,9], both directly (e.g., during work and recreational outdoor activities) and indirectly (e.g., ingesting contaminated seafood). In this regards, dinoflagellates and cyanobacteria are the most important representatives of harmful classes in marine and freshwater ecosystems, respectively.

Among dinoflagellates, the majority of the known *Alexandrium* species are toxic [10]. The toxic compounds produced belong to saxitoxins, spirolides, goniodomins and gymnodimines, but the most important in terms of impact on humans are saxitoxins [11,12,13]. Saxitoxin and its analogues (paralytic shellfish toxins, PSTs) are neurotoxic alkaloids mainly responsible for paralytic shellfish poisoning (PSP), one of the most serious bio-intoxications [14,15]. PSP outbreaks have harmful effects on human health as well as important economic implications for aquaculture and shellfish farms, the impairment of tourism and recreational activities, alteration of marine trophic structure and the death of marine mammals, fishes and seabirds [11]. Humans can contract PSP by ingesting seafood, mainly shellfish that have accumulated PSTs in their tissues. The member states of the European Union must ban mussel harvesting if PSTs in shellfish exceed the limit of 800 µg STXeq kg^−1^ in any edible part (2004/853/EC).

Since the beginning of 2000, eight closures and marketing blocks of farming areas (closure period up to twenty-seven days in the Gulf of Olbia in 2003, [16]) have occurred along the coast of Sardinia, the second largest Mediterranean island (western Mediterranean Sea, Italy), making this region the most affected by PSP in Italy [16,17,18]. The main organisms responsible for these events were *Alexandrium pacificum* Litaker (ex *A. catenella* (Whedon and Kofoid), Balech), and *A. minutum* Halim [16,19]. These two species bloomed singularly or together in mixed blooms when mussel contaminations were detected. More recently (2011), mixed blooms of *A. minutum* and *A. pacificum* were also observed along the Sicilian coasts (Syracuse Bay), in addition to the recurrent, seasonal blooms of *A. minutum* already recorded since the beginning of the 2000s [20]. However, only two cases of shellfish farm closures in Sicily are known, in winter 2007 and autumn 2008, (unpublished data of the RF-IZI-2008-1139874 project), due to local difficulties in establishing a regular control plan for mussels.

Cyanobacteria are known to produce harmful major hepato- and neuro-toxins (microcystins, anatoxins, cylindrospermopsins, saxitoxins, β-*N*-methyl-amino-l-alanine), and a lot of minor toxins (e.g., anabaenopeptilides, anabaenopeptins, cyanopeptolins, aeruginosins, microginins, microviridins, nostophycins) [21,22,23,24], with various enzyme inhibition roles in cellular metabolism. Cyanobacteria can also produce PSTs [25]; furthermore, the majority of them appears involved in the β-*N*-methylamino-l-alanine (BMAA) production [26], a potent, not yet well-investigated neurotoxin. Originally discovered in cyanobacterial species symbionts of Cycadaceae, but then detected in 95% of cyanobacteria [27,28], BMAA, like its isomer DABA (2,4-diaminobutyric acid dihydrochloride), is a non-proteic amino acid associated with neurodegenerative syndromes, particularly amyotrophic lateral sclerosis and Alzheimer’s disease. BMAA may be misincorporated by nerve cells in place of serine in proteins, leading to a misfolding of the protein structure and giving rise to neurodegeneration and the death of brain motor neurons. This mechanism of bio-incorporation was suggested early [29].

Particular attention has to be paid to BMAA co-presence with microcystins (MCs) in environmental matrices, due to the ability of the latter to generate oxidative stress to DNA and neurological damage in the hippocampus, with amnesic effects [30]. MCs are a family of more than 90 potent eptapeptide hepatotoxins [21], and are the most frequent freshwater cyanotoxins recognized worldwide [31], including in the Mediterranean region [32,33].

One of the main concerns connected to cyanotoxins is in regard to their presence in water for drinking [34,35]. About 90% of the drinking water in Sardinia is derived from artificial lakes, which are mainly eutrophic and dominated by cyanobacteria. Cyanotoxins have been detected both in waters and scums in Sardinian artificial lakes since the 1980s [33], and a strong positive relationship between MCs and eutrophication was assessed more recently [36].

This study reports new data on PST profiles, cell morphology and the genetics of *Alexandrium* strains (*A. minutum*, *A. pacificum*, *A. tamarense*) obtained from several sites along the Sardinian and Sicilian coasts. Further, the study includes the first data on BMAA concentrations in addition to MCs data in natural samples from a Sardinian artificial lake (Lake Bidighinzu), whose waters are used for drinking. BMAA and MCs were also analysed in cyanobacterial strains obtained from samples collected in the same lake. The aim of this study was to give novel information on the toxicity of both dinoflagellates and cyanobacteria, to contribute to the knowledge of the toxicity and biodiversity of these two toxic groups in the Mediterranean region.

## 2. Materials and Methods

### 2.1. Dinoflagellates

#### 2.1.1. Sampling and Cell Cultures

**Figure 1:**
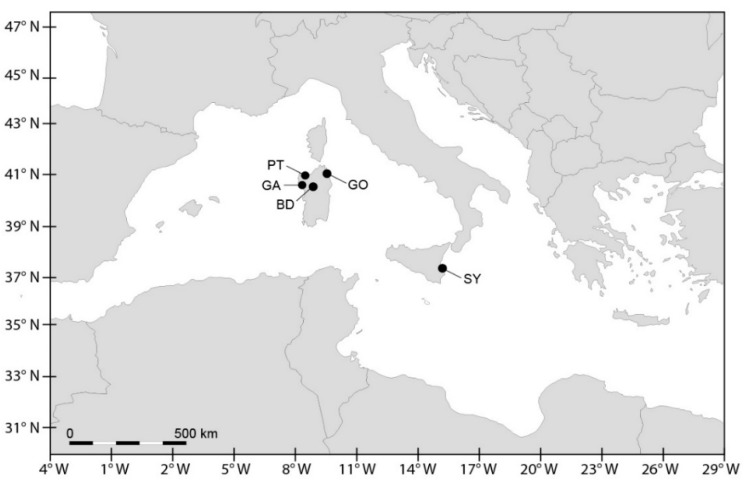
Location of the study area and sampling sites. (Lake Bidighinzu = BD; Gulf of Olbia = GO; Porto Torres harbour = PT; Gulf of Alghero = GA; Syracuse bay = SY).

In the context of programmes for the monitoring and management of HABs (Projects MiPA 5C8—Fisheries and Aquaculture, Strategy EVK3-CT-2001-00046, SEED GOCE-CT-2005-00387, PO Sardegna FSE 2007-2013-L.R.7/2007, regional plan of surveillance, vigilance and sanitary control of production and marketing of molluscs of Sardinia), water sampling was carried out in the period from 2001 to 2013 in the Olbia harbour (Gulf of Olbia, Tyrrhenian Sea, Sardinia, Italy; GO) and in the Syracuse harbour (Syracuse bay, Ionian coast, Sicily, Italy; SY) (Figure 1). Sardinia is among the most relevant shellfish producers in Italy with 9753 tons y^−1^, one third of which derives from the Gulf of Olbia. Shellfish production in Sicily reaches 625 tons y^−1^ [37]. Surface (−0.30 m) water samples were collected at 2–4 sampling points at each site. During some occasions, vertical net samples (mesh 10 µm), from about −2 m to the surface, and a higher number of stations were sampled. These extra samples were linked to special situations in the sampling sites (e.g., PSTs > 800 µg STXeq kg^−1^) and/or the special needs of the researchers (e.g., the objectives of specific projects). Surface water samples were also collected in the Porto Torres harbour (Gulf of Asinara, Sardinia, Italy; PT) at one sampling point along an annual cycle in 2002. A single sediment sampling was carried out by a scuba diver using cylindrical plastic corers (20 cm long with a diameter of 5 cm) in May 2010, in the Gulf of Alghero (Sardinian Sea, Sardinia, Italy; GA).

Twelve cell cultures of *Alexandrium* species were obtained from the above sites, eleven by the isolation of vegetative cells from water samples, and one by the germination of resting cysts from sediment samples (Table 1). Cells and cysts were isolated with glass micropipettes or a micromanipulator Narishige and then transferred into tissue culture multiplates filled with L1, F20 or F2 medium, without the addition of silicate. Cultures were grown at (17–20) ± 1 °C with a photoperiod of 12:12 light:dark cycle and irradiance of 100 µmol photons m^−2^ s^−1^.

#### 2.1.2. Morphological Analysis

The morphology of alive and fixed cells (Lugol’s solution, 2% final concentration) from cultures was analysed using inverted Zeiss microscopes (Axiovert 10, 25, 35 and 200; Carl Zeiss, Oberkochen, Germany) at 400× and 1000× magnifications, equipped with digital cameras (Spot Flex, Spot imaging, Sterling Heights, Michigan, USA and Zeiss Axiocam, Carl Zeiss, Oberkochen, Germany). Plate patterns were studied following Balech (1995) [38] after staining with Calcofluor white (Fluorescent Brightener 28, Sigma, Steinheim, Germany) and observation under UV epifluorescence (filter set 487902, Zeiss filters, Carl Zeiss, Oberkochen, Germany) [39]. Cell sizes were determined using calibrated eyepieces and the AxioVision 3.1 software (Carl Zeiss Vision, GmbH, München-Hallbergmoos, Germany).

#### 2.1.3. Toxin Analysis

PST analysis was conducted on cultures in the exponential growth phase, within three months from the isolation of vegetative cells. Exact volumes and cell counts per culture were duly recorded for further calculations. PST analysis was conducted by high performance liquid chromatography (HPLC) with pre-column oxidation and fluorescence detection (FLD) [40] for the *Alexandrium* strains CNR–ACATSRA4, UNISS3, UNISS4, CNR–AMID6, CNR–AMISY1 and UNISS5, and with HPLC–FLD post-column oxidation [41,42,43] for the *Alexandrium* strains CNR–ACATS2, CNR–ACATC2, CNR–AMIA2PT, CNR–AMIA4, CNR–AMIA5, and CNR–ATA6PT.

Aliquots of the cultures of the *Alexandrium* strains CNR–ACATSRA4, UNISS3, UNISS4, CNR–AMID6, CNR–AMISY1 and UNISS5 were centrifuged at 4500 rpm to obtain a concentrated algal pellet that was subsequently extracted using a maximum of 5 mL acetic acid 0.1 M. The acidic pellet was sonicated (Ultrasonic^®^ Liquid Processor Model XL2020, Heat Systems Inc., New York, NY, USA) for 30 min in pulse mode, to break the cells, and then centrifuged again at 4500 rpm for 10 min. Aliquots of 1 mL of the supernatant were further cleaned up using C18 SPE cartridges (Supelclean LC18, 500 mg/3 mL, Supelco, Bellefonte, PA, USA) and eventually 10-fold concentrated by evaporation under stream of nitrogen using mild heating. Analysis was performed using both peroxide and periodate oxidation steps. The method allowed this study to quantify individual PSTs, except for the epimeric pairs (for example GTX1 and 4), which form identical oxidation products and cannot be separated [44].

Toxins were quantified against linear calibrations of all currently available PST certified reference standards. No ion exchange fractionation was undertaken prior to the quantification of the *N*-hydroxylated toxins, such as GTX1 and 4. The limit of quantification (LOQ) of the analytical method for each toxin (STX, GTX1 and 4, GTX2 and 3, GTX5, C1/2, neoSTX, dcSTX, dcGTX2 and 3) was in the range of 0.02–0.15 pmol injected on the column. The limit of detection (LOD) was assumed to be one-third of the limit of quantification.

Aliquots of the cultures of *Alexandrium* strains CNR–ACATS2, CNR–ACATC2, CNR–AMIA2PT, CNR–AMIA4, CNR–AMIA5, and CNR–ATA6PT were sonicated with 0.1 M acetic acid. After centrifugation, the supernatants were treated for SPE clean-up according to [45] and eluates were injected into a HPLC system. The LC determination of PSTs was carried out using ion-pair elution with sodium hexanesulfonate and sodium heptanesulfonate, and post-column oxidation with periodic acid and fluorescence detection based on [41,42,43]. Toxins were quantified using the reference standards available at the time.

#### 2.1.4. Biomolecular Analysis

The genomic DNA of *A. tamarense* UNISS5 was extracted from approximately 10–20 mL of culture in logarithmic growth phase. Cells were harvested by centrifugation at 3000 rpm for 15 min. The pellet was transferred to a 2 mL Eppendorf tube and centrifuged again at 10,000 rpm for 5 min. Total genomic DNA was extracted from the resulting pellet using a DNeasy Plant Kit (Qiagen, Valencia, CA, USA), according to the manufacturer’s instructions. The extracted DNA was immediately frozen at −80 °C. *A. minutum* UNISS3 and UNISS4 were analysed applying the single-cell PCR method [50]. Individual cells were isolated under an inverted microscope (Zeiss Axiovert 25, Carl Zeiss, Oberkochen, Germany) with a glass pipette and transferred to 200 µL PCR tubes containing 5 µL of lysis solution (0.005% SDS and 400 ng µL^−1^ Proteinase K). Tubes were briefly centrifuged to ensure that the cells were at the bottom. Tubes were stored at −20 °C. Lysis involved the thawing of tubes and the freezing at −80 °C for at least 10 min, incubation at 60 °C for 30 min and finally at 95 °C for 10 min. The lysates were used immediately, or stored at −80 °C until used for the PCR.

The primers D1R and D2C [51] were used to amplify the large subunit LSU rRNA gene and the primers ITSF01 and ITS4 [52,53] were used to amplify the 5.8S rDNA and internal transcribed spacers (ITS1 and ITS2 regions). The PCRs for *A. tamarense* UNISS5 were carried out in 25 µL reactions containing 1 µL of DNA extract, 0.625 µL of each primer (10 µM), 1.5 µL of dNTPs (200 µM of each), 1.5 µL of MgCl_2_ (25 mM), 2.5 µL of 10× PCR buffer, and 0.125 µL of Taq DNA polymerase (Qiagen). The PCRs for *A. minutum* UNISS3 and UNISS4 were carried out in 50 µL reactions containing 1 µL of DNA extract, 4 µL of each primer (10 µM), 1 µL of dNTPs (200 µM of each), 1 µL of MgCl_2_ (25 mM), 5 µL of 10× PCR buffer and 0.25 µL of Taq DNA polymerase (Qiagen). PCR cycles included one initial step at 95 °C for 5 min followed by 40 cycles at 95 °C for 20 s, 55 °C (for primers D1R and D2C) or 53 °C (for primers ITSF01 and ITS4) for 30 s and at 72 °C for 1 min, followed by a final extension at 72 °C for 10 min. Aliquots of the PCR products were electrophoresed in 1.2% agarose gels. The remaining product was stored at 4 °C until sequenced.

The sequences obtained for UNISS3, UNISS4 and UNISS5 were aligned with those obtained from GenBank using the MAFFT v.6 program) [54] under FFT–NS–i (slow; iterative refinement method). Phylogenetic relationships were done with the maximum-likelihood (ML) method and the GTRGAMMA evolution model on Randomized Axelerated Maximum Likelihood (RAxML) v. 7.0.4 [55]. Repeated runs on distinct starting trees were carried out to select the tree with the best topology (the one with the greatest likelihood of 1000 alternative trees). Bootstrap ML analysis was done with 1000 pseudo-replicates and the consensus tree was computed with Mr. Bayes [56]. All analyses were done through the freely available University of Oslo Bioportal [57].

### 2.2. Cyanobacteria

#### 2.2.1. Sampling

Sampling was conducted monthly from April 2014 to March 2015 at a single station close to the deepest part (depth: 25 m) in Lake Bidighinzu (North-Western Sardinia; Figure 1). Water samples were collected using a Niskin bottle along a vertical profile from the surface (−0.5 m) and at depths of −1, −2.5, −5, −7.5 and −10 m. From each depth, a sample (100 mL) was fixed in the field using Lugol’s solution for cyanobacteria counts and species identification and another sample (1000 mL) was collected for quantitative toxin analysis. Samples for toxin analysis were stored at −20 °C until processing.

#### 2.2.2. Cyanobacterial Abundance and Species Composition in Natural Samples

Phytoplankton samples were analysed using Utermöhl’s technique [58]. Cyanobacteria cell density was determined microscopically from subsamples (5–10 mL) of the fixed samples, using an inverted microscope (Zeiss, Axiovert 10, Carl Zeiss, Oberkochen, Germany) at 200× and 400× magnifications, based on cell counts from an appropriate number of fields. Species were identified from alive and fixed samples according to the available taxonomic guides [59,60,61,62,63]. Cyanobacteria data were transformed (weighted mean) to represent integrated samples in the photic zone (Zeu = 2.5 times the Secchi disk depth; [64]).

#### 2.2.3. Cell Cultures

One strain of *Microcystis aeruginosa* (UNISS12) and one of *Dolichospermum flos-aquae* (UNISS13) were obtained by isolation of vegetative cells (respectively, one colony and one trichome) from water samples collected in Lake Bidighinzu.

Specimens were isolated with glass micropipettes and transferred into tissue culture multiplates filled with BG11 medium. Cultures were grown at 21 ± 1 °C with a photoperiod of 16:8 light:dark cycle and irradiance of 50–70 µmol photons m^−2^ s^−1^.

#### 2.2.4. Toxin Determination

Enzyme-linked immunosorbent assay (ELISA) detected BMAA, MCs and nodularins (NOD) in natural water samples and cultures. The samples were analysed using ELISA kits from Abraxis (Microcystins ADDA–ELISA Microtiter Plate for MCs and BMAA kit), after a slow freeze–thaw cycle followed by ultrasonic treatment (ELMA S 10, Elmasonic, Singen, Germany), according to the manufacturer’s protocols. Despite initial criticisms of its reliability [65], the Abraxis BMAA kit was positively validated in recent studies [66]. The absorbance was read using a Mini photometer (model 6+, Metertech Inc., Taipei, Taiwan). The results were expressed, respectively, as µg L^−1^ of MC–LR equivalents, as indicated by the manufacturer, and were reported in the text as MCs, and as µg L^−1^ for BMAA. Samples were considered positive when the MCs and BMAA concentrations were higher than the lowest detection limit (LDL; 0.10 µg L^−1^ and 4 µg L^−1^, respectively). During this study, toxin analyses were carried out on the water samples collected at the two depths of cyanobacteria abundance maxima in each date of sampling, and reported as average data.

## 3. Results

### 3.1. Dinoflagellates

#### 3.1.1. Morphology of *Alexandrium* Strains

**Figure 2:**
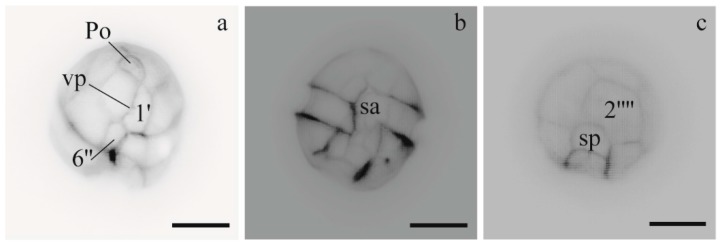
Light micrographs of *Alexandrium minutum* cells (UNISS3 and UNISS4 strains), stained with Calcofluor and observed using UV fluorescence. (**a**,**b**) apical and ventral views showing the ventral pore (vp) in the 1′ plate and the wide anterior sulcal plate (sa); (**c**) antapical view showing the 2′′′′ plate. Scale bars 10 μm.

*A. minutum* cultures exhibited single cells with a ventral pore in the 1′ plate. UNISS3 and UNISS4 showed the same plate pattern with the 1′ plate truncated anteriorly and posteriorly, the anterior sulcal plate wide and the 2′′′′ plate of type B (transversal axis longer than the longitudinal axis) (Figure 2). Cell sizes ranged from 20 µm to 26.3 µm in length (mean: 22.3; *n* = 40), and from 17.5 µm to 25 µm in width (mean: 20.6; *n* = 40). *A. minutum* CNR–AMIA2PT cells were slightly smaller than the other specimens. The size range was 16.9 µm to 26.5 µm (mean = 21 µm; *n* = 20) and 15 µm to 19.8 µm (mean = 17.8 µm; *n* = 20), respectively for length and width.

**Figure 3:**
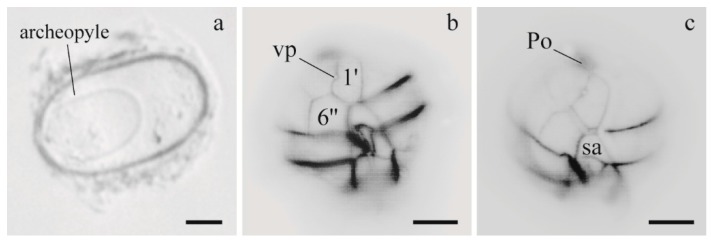
Light micrographs of *Alexandrium tamarense* cyst and cells (UNISS5 strain), stained with Calcofluor and observed using UV fluorescence. (**a**) empty cyst showing the shape of archeopyle; (**b**,**c**) ventral views showing the ventral pore (vp) in the 1′ plate, the wide 6′′ plate and the connection between the 1′ plate and apical pore plate (Po). Scale bars 10 μm.

*A. tamarense* UNISS5 culture was derived from the germination of a greyish, ellipsoidal cyst (50 µm long and 28 µm wide), with a granular content and a yellow accumulation body. The archeopyle was oval and extended for a half-length of the cyst (Figure 3). *A. tamarense* UNISS5 and CNR–ATA6PT cells were mainly single or in chains of two and had a ventral pore in the 1′ plate. Cells of both strains shared the same plate pattern, characterized by a large 6′′ plate and 1′ plate in direct contact with Po (Figure 3). The cell size of UNISS5 ranged from 22.5 µm to 32.5 µm (mean = 27.7 µm; *n* = 20) in length and from 22.5 µm to 33.8 µm (mean = 28.7 µm; *n* = 20) in width. Also, the cell size of CNR–ATA6PT was very similar, with a mean length and width of 27.8 µm and 28.3 µm, respectively.

Specimens of *A. pacificum* (CNR–ACATS2 and CNR–ACATC2) from cultures exhibited the same cell size and pattern of thecal plates of those described from the field in the Gulf of Olbia [67]. Additionally, the isolates from the Sicily culture (CNR–ACATSRA4) displayed similar morphological traits with a size range of 27.7–36.6 µm for length and 28.5–37.4 µm for width.

#### 3.1.2. Toxins of *Alexandrium* Strains

**Figure 4:**
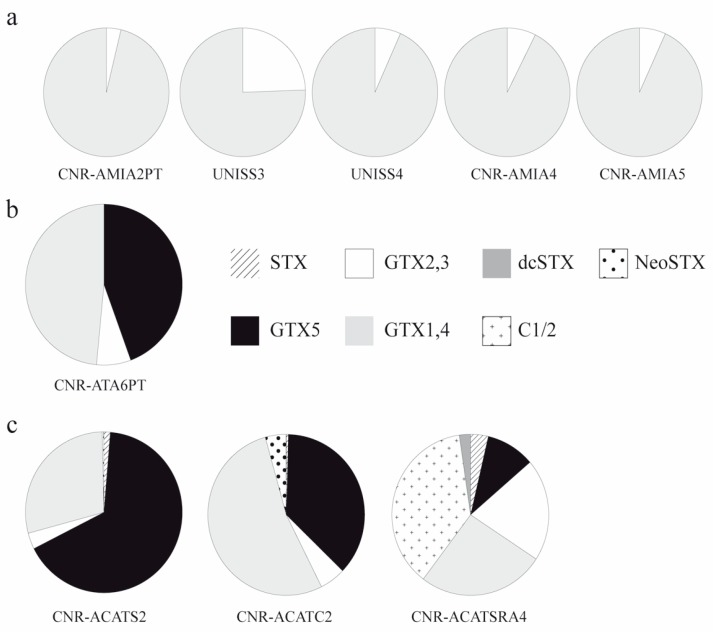
Percentage composition of PST toxins in the analysed strains. **a** = *Alexandrium minutum* strains; **b** = *A. tamarense* strain; **c** = *A. pacificum* strains.

Strains of *A. minutum* from Sardinian sites (CNR–AMIA2PT, UNISS3 and UNISS4) and two strains (CNR–AMIA4 and CNR–AMIA5) from SY were found to be toxic (Table 2). These strains revealed higher concentrations of GTX1 and 4 than GTX2 and 3 (Figure 4). CNR–AMIA2PT was the most toxic strain, followed by the CNR–AMIA4 and CNR–AMIA5 strains, and UNISS3 and UNISS4 strains (Table 2).

*A. tamarense* CNR–ATA6PT showed moderate toxicity, especially due to GTX5 and GTX1 and 4 (Table 2, Figure 4). No toxicity (<LOD) was found for *A. tamarense* UNISS5 (Table 2).

*A. pacificum* CNR–ACATS2 and CNR–ACATC2 showed a total toxicity up to 17.811 fmol cell^−1^, which was markedly higher than the total toxicity found for *A. pacificum* CNR–ACATSRA4 (Table 2). GTX1 and 4 and GTX5 were the most abundant toxins for *A. pacificum* CNR–ACATS2 and CNR–ACATC2 (Table 2, Figure 4). STX and GTX5 values were notably higher for CNR–ACATS2 than CNR–ACATC2. Rather, GTX2 and 3, GTX1 and 4 and NeoSTX were higher for CNR–ACATC2 than CNR–ACATS2. GTX1 and 4 and C1/2 prevailed in the toxin profile of *A. pacificum* CNR–ACATSRA4 (Table 2; Figure 4).

#### 3.1.3. Genetics of *Alexandrium* Strains

**Figure 5:**
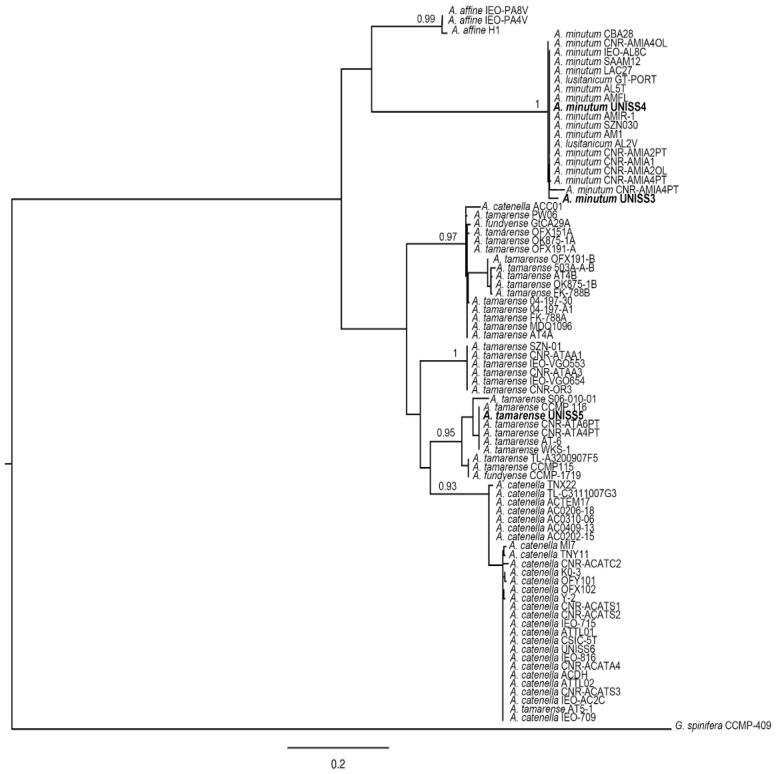
Maximum likelihood (ML) tree of the 5.8-ITS rRNA sequences of selected *Alexandrium* species (Table S1). Values at the branch nodes indicate ML bootstrap support (BS). Only BS values >50 are shown. The *Gonyaulax spinifera* sequence was used as an outgroup. The organisms sequenced in this study are shown in bold.

**Figure 6:**
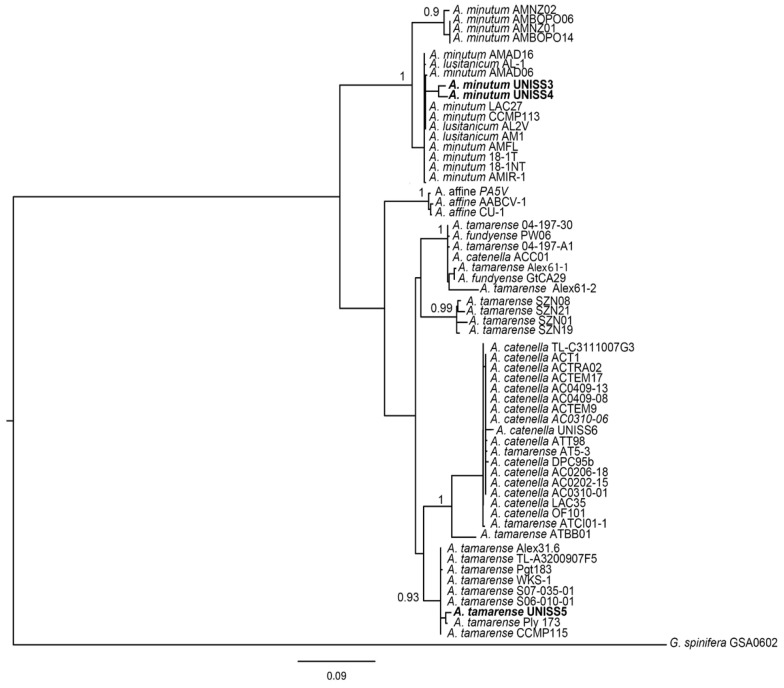
Maximum likelihood (ML) tree of the LSU rRNA sequences of selected *Alexandrium* species (Table S2). Values at the branch nodes indicate ML bootstrap support (BS). Only BS values >50 are shown. The *Gonyaulax spinifera* sequence was used as an outgroup. The organisms sequenced in this study are shown in bold.

*A. tamarense* UNISS5 and CNR–ATA6PT grouped with the Group III and *A. minutum* UNISS3 and UNISS4 with the Global Clade (Figure 5 and Figure 6). The analyzed isolates showed identities of 99% or 100% with the *Alexandrium* sequences from GenBank for the respective clades of belonging.

Data on the genetic analyses of *A. pacificum* CNR–ACATS2, CNR–ACATC2, CNR–ACATSRA4, *A. tamarense* CNR–ATA6PT, *A. minutum* CNR–AMIA2PT, CNR–AMID6, CNR–AMISY1, CNR–AMIA4 and CNR–AMIA5 have already been reported [20,47,48,49]. All sequences used in the trees are listed in Supplementary Materials (Tables S1 and S2).

### 3.2. Cyanobacteria

#### 3.2.1. Species Composition and Cell Abundance

**Figure 7:**
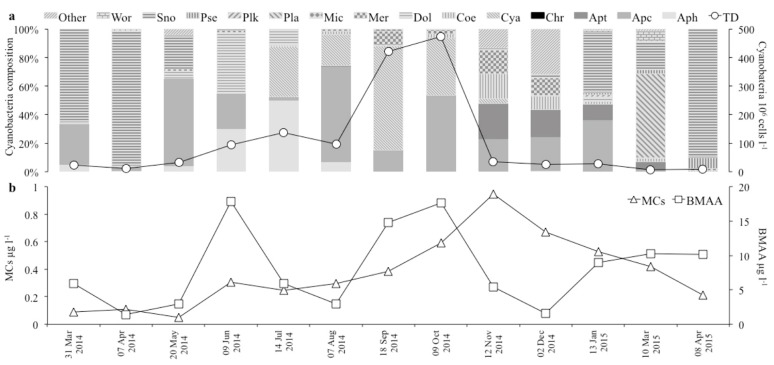
Temporal dynamics of (**a**) cyanobacteria composition at the genus level (left axis) and cell density (right axis) and of (**b**) microcystins (left axis) and BMAA (right axis) concentrations. (TD = cyanobacterial total cell density; Aph = *Aphanizomenon*; Apc = *Aphanocapsa*; Apt = *Aphanothece*; Chr = *Chroococcus*; Cya = *Cyanocatena*; Coe = *Coelosphaerium*; Dol = *Dolichospermum*; Mer = *Merismopedia*; Mic = *Microcystis*; Pla = *Planktolyngbya*; Plk = *Planktothrix*; Pse = *Pseudanabaena*; Sno = *Snowella*; Wor = *Woronichinia*; Other = unidentified cyanobacteria; BMAA = β-*N*-methylamino-l-alanine; MCs = microcystins).

The observed twenty-two taxa of cyanobacteria belonged to fourteen genera, predominantly Chroococcales (Table 3). The total cell density (TD) varied between about 7 × 10^6^ cells L^−1^ in March 2015 and 475 × 10^6^ cells L^−1^ in October 2014 (Table 3; Figure 7). Higher TD values were observed from summer to autumn, particularly from September to October. Species composition varied strongly during the year (Table 3; Figure 7). The species responsible for the peaks were, in hierarchical order, *Cyanocatena* sp., *Aphanocapsa incerta* (Lemmermann) G. Cronberg and Komárek and *Merismopedia tenuissima* Lemmermann in September and *A. incerta*, *Cyanocatena* sp. and *Aphanocapsa* sp. in October.

#### 3.2.2. Toxins and Dominant Species

The MC and BMAA presence was assessed in all the analysed samples. The MCs varied from 0.08 µg L^−1^ to 0.945 µg L^−1^ in March and November 2014, respectively (Table 3).

The MCs increased progressively from spring to autumn, achieving the maximum in November, a month after the maximum TD. *Aphanothece* sp., *Aphanocapsa* sp. and *Coelosphaerium* sp. were the dominant species (accounting for about 64% of TD) during MCs maximum (Figure 7).

The BMAA varied from 1.427 µg L^−1^ to 17.848 µg L^−1^, respectively, in April 2014 and June 2014 (Table 3). The BMAA peaks were in spring (June 2014 and April 2015), late summer (September), autumn (October), when TD was highest, and in the winter months (January and March 2015), coinciding with different cyanobacteria species compositions. Specifically, *Dolichospermum flos-aquae* (Brébisson ex Bornet and Flahault) P. Wacklin, L. Hoffmann and J. Komárek, *Aphanizomenon flos-aquae* (Ralfs ex Bornet and Flahault) and *Aphanocapsa* sp. were the dominant species in June (accounting for about 91% of TD) and *Snowella lacustris* (Chodat) Komárek and Hindák in April 2015 (accounting for about 90% of TD); *Cyanocatena* sp., *M. tenuissima* and *A. incerta* in September (accounting for about 92% of TD); *A. incerta*, *Cyanocatena* sp. and *Aphanocapsa* sp. in October (accounting for about 94% of TD); *S. lacustris*, *Aphanocapsa* sp. and *Aphanothece* sp. in January (accounting for about 89% of TD) and *Planktolyngbya* sp. and *S. lacustris* in March 2015 (accounting for about 79% of TD) (Figure 7)*.*

MCs and BMAA were found in the cell cultures of *D. flos-aquae* and *Microcystis aeruginosa* (Kützing) Kützing, with concentrations of 0.509 µg L^−1^ and 0.097 µg L^−1^ for MCs, and 1.299 µg L^−1^ and 19.289 µg L^−1^ for BMAA, respectively.

## 4. Discussion

### 4.1. Dinoflagellates

The *Alexandrium* species substantially contributes to HAB events worldwide. A great richness of species appears in the Mediterranean Sea, probably due to the notable level of taxonomic scrutiny [11,47]. The ecological and economic impacts of the *Alexandrium* species increased along the Mediterranean coasts during the last three decades, leading to numerous and detailed studies on this topic [16,19,20,67,68,69,70,71,72,73,74,75]. The present work offers new information on toxin profiles, cell morphology and the genetics of *A. minutum*, *A. pacificum* and *A. tamarense* strains.

Identification of the *Alexandrium* species is based on morphological criteria (thecal plate arrangement; [38,76]), but in some cases, the subtle morphological characteristics used for classification are not easily resolved (cell size, shape, chain formation, theca ornamentation, cingular and sulcal excavation, presence of sulcal lists, shape of the apical pore complex, shape of 1′ and 6′′ plates, and of sulcal plates). Recently, various molecular techniques have been employed using different genetic markers to support the morphological identification of the species [47,49,51,77,78,79,80,81]. The taxonomic architecture of *Alexandrium* genus is still debated, especially for specific groups of species [82,83,84]. Focusing on the three *Alexandrium* species considered in this work, *A. minutum* groups into the “*A. minutum* species complex”, and *A. pacificum* and *A. tamarense* derived from the recent taxonomic nomenclatural revision of the “*A. tamarense* species complex” [83]. The “*A. tamarense* species complex” included three morphospecies (*A. tamarense*, *A. catenella* and *A. fundyense*) and five ribotypes or genetic lineages that were initially geographically named [78,85,86] and then renamed as groups I, II, III, IV and V [83]. John et al. (2014) [83] found that each group represented a distinct cryptic species and, accordingly, species names were assigned for the groups, namely *A. fundyense* (group I), *A. mediterraneum* (group II), *A. tamarense* (group III), *A. pacificum* (group IV), and *A. australiense* (group V). Furthermore, the proposal to reject the name *Gonyaulax catenella* (*Alexandrium catenella*) [82] was criticized [84] and the debate is still in course.

Species identification is not sufficient to obtain information on the potential harmfulness of the species, since toxic and non-toxic strains can be found within the same species [11], as also revealed during this study. Indeed, toxicological analyses are required [87,88]. The genes responsible for PST synthesis have been identified for several *Alexandrium* strains [89,90]. *Alexandrium* strains may have positive or negative results for these genes, also within the same species, as recently ascertained for Mediterranean strains of *A. minutum* [48]. Furthermore, environmental factors (e.g., nutrients, CO_2_, salinity, temperature) influence PST content [91,92,93,94] and PST-producing pathways at the molecular level in *Alexandrium* strains [95]. Since PSTs include compounds whose toxic potency varies by orders of magnitude due to certain minor structural differences [91,92], the cellular toxicity of PST producers is not only determined by their PST content, but also by the relative composition of the different PST analogues [96]. Usually, a PST producer synthesizes a characteristic suite of toxins made up of several PST analogues [11,14]. In addition, PST content varies depending on growth phases as observed in batch cultures, with maxima usually found in exponential phases and under P-limitation [11]. Regarding food chains, differences in PST profiles can be found between natural phytoplankton samples containing *Alexandrium* toxic species and samples of shellfish that filtered those waters and accumulated toxins in their tissues [72]. Toxins, once assimilated by filtering organisms, can be transformed leading to changes in net toxicity. Toxin biotransformation varies greatly among species. Also, toxin accumulation shows interspecific differences linked to diverse sensitivity to toxins, determined from neurological, physiological, and behavioural responses [97].

The *A. pacificum* strains were the most toxic and their profiles were the richest in PST analogues among all the analysed strains in this study. Profiles varied among the *A. pacificum* strains and resulted in differences characterized by a prevalence of GTX5, GTX1 and 4 and C1/2 on the other analogues (i.e., GTX2 and 3, STX, deSTX and NeoSTX). Tatters et al. (2013) [92], who investigated the effects of nutrients and global climate change on the toxicity of an *A. pacificum* strain isolated from Southern California, reported a predominance of GTX5 and C1/2 in the control, and an enhancement of GTX1 and 4 concentrations at high CO_2_ conditions. Krock et al. (2007) [98] reported that C1/2, B1 and GTX1 and 4 comprised >90% of the total PST content on the molar basis in a strain of *A. pacificum* from Chile. Murray et al. (2011) [99] analysed three Australian strains of *A. pacificum* and reported similar amounts of analogues of PSTs and relatively similar toxin profiles, producing primarily C1/2 and GTX1 and 4 analogues.

*A. pacificum* has already been reported in the Mediterranean Sea [47,48]. All Mediterranean isolates of *A. pacificum* showed the same sequence of the Japanese ITS–5.8S rRNA genes, supporting the hypothesis of an alien character of the species in the Mediterranean Sea, probably mediated by human activities with the transport of vegetative or resting forms [47]. However, Masseret et al. (2009) [80] argued that *A. pacificum* might have been part of the hidden flora (as resting cysts or in low quantities) in the Mediterranean Sea and its recent expansion could be favoured by changing environmental conditions (e.g., related to local anthropogenic pressure or global climate change). Collos et al. (2009) [100] supported this hypothesis considering long-term trends of temperature, salinity, nutrients and modification in phytoplankton composition. In this regard, integrated studies on the planktonic and benthic compartments, on the vegetative and resting stages, and on long-term series of data allow us to acquire more detailed information on the geographical distribution of harmful algal species (HAS) [101,102,103,104]. To support Masseret’s hypothesis, one can report the case of a strain of *A. pacificum* (UNISS6) obtained by the germination of a cyst isolated from sediments collected in the Santa Giusta Lagoon (Western Sardinia) [103]. The phytoplankton of this lagoon (research site belonging to the Italian long-term ecological research network) has been studied from 1990 to date, but *A. pacificum* cells have never been observed in the 900 analysed samples [105].

In contrast to the proven toxicity of *A. pacificum*, *A. mediterranium* U. John (group II) strains were found to be non-toxic in the Mediterranean Sea. Recently, several Mediterranean *A. tamarense* (group III) strains were also identified. This study reports the toxin profile of one of two strains from Porto Torres (North Western Sardinia) attributed by Penna et al. (2008) [47] to group III, documenting the presence of toxic strains of *A. tamarense* also in the Mediterranean Sea. Additionally, the non-toxic strain UNISS5 reinforces the reports on the presence of *A. tamarense* and that the same species can be toxic or non-toxic. The alignment of *A. tamarense* UNISS5 with *A. tamarense* WKS 1 of the Pacific and with the European IEO–PE1V and CCMP 116 of the North Atlantic, respectively non-toxic and toxic, confirms this affirmation. The toxin profile of *A. tamarense* CNR–ATA6PT cannot be compared with others from the same geographical area because it is the first record for the Mediterranean. Toxin analysis of this strain, as for other strains considered in this work, was carried out at the beginning of the 2000s. Toxin data obtained at that time should be confirmed in the future using more recent and proven methods and instruments than those used in 2002–2003. The assessed profile of *A. tamarense* CNR–ATA6PT strain, with the most relative abundance of GTX1 and 4 and GTX5 and lesser amounts of GTX2 and 3, was consistent with those reported in other studies [106,107]. Zou et al. (2014) [107] analysed 67 Chinese strains, and found that the toxins with the highest concentration in the profile were C1/2, GTX1 and 4 and neosaxitoxin. However, the toxin profiles were atypical and C1/2 toxins were not detected in some strains. Hold et al. (2001) [108] investigated the influence of natural bacteria in the PST production of *A. tamarense* cultures. They found that GTX1 and 4 were the dominant toxins in both the free-bacteria and control cultures (i.e., without antibiotic treatment).

*A. minutum* UNISS3 and UNISS4 responded fully to the diacritical characters of *A. lusitanicum* Balech, currently considered as a morphotype within the morphological variability of *A. minutum* [109,110,111]. The *A. minutum* UNISS3, UNISS4 and CNR–AMIA2PT grouped with the Global Clade that includes both toxic and non-toxic strains [10]. The *A. minutum* UNISS3, UNISS4 and CNR–AMIA2PT revealed higher concentrations of GTX1 and 4 than GTX2 and 3, in agreement with data reported for other Mediterranean areas [110]. Stüken et al. (2015) [112] reported three groups of PST profiles in *A. minutum* strains. One group comprised strains isolated from different locations in the Mediterranean Sea and the Atlantic Ocean, characterized by the presence of GTX1 and 4 and GTX2 and 3 for the majority of the analysed strains, similar to the results of the present study. Another group comprised a strain (VGO663), isolated from Sardinia, characterized by no PST detection, similarly to CNR–AMID6 and CNR–AMISY1 strains. Penna et al. (2015) [48] reported the presence of the major gonyautoxins GTX1 and 4 in 88% of the Mediterranean strains studied, GTX1 and 4 together with small amounts of GTX2 and 3 in 16% of the strains, and very few strains did not produce any toxin in detectable amounts. Perini et al. (2014) [93] reported that in four Mediterranean *A. minutum* strains, the composition of toxins was related to the phenotypic trait and the toxin amounts were variable among strains, even when they were from the same geographical area. *A. minutum* produced primarily GTX1 and 4. GTX4 represented nearly 98% of the total cell toxin content, followed by GTX1, GTX2 and GTX3 in *A. minutum* strain isolated from the Gulf of Trieste in 1993 [17]. Finally, the GTX 1, 2, 3 and 4, with the dominant epimer pairs GTX1 and 4, were detected in both axenic and control cultures of *A. minutum* (reported as *A. lusitanicum*). This implies that the removal of bacteria did not inhibit toxin production by this dinoflagellate nor change the toxin profile [108].

### 4.2. Cyanobacteria

Cyanobacteria are likely among the most widespread, abundant, and ancient organisms on Earth. Interest in this group is growing globally together with the increase of the geographical distribution, frequency and extent of their harmful blooms (CyanoHABs; [113]), which are expected to further increase due to climate change [7,114]. Forty species within ten genera are mainly responsible for toxic CyanoHABs [115,116,117]. It is estimated that more than 50% of CyanoHABs are toxic, causing economic losses in addition to health problems for ecosystems and humans [118,119,120].

Microcystin, nodularin and cylindrospermopsin are among the most frequently occurring cyanotoxins in surface and drinking water [34,116]. Messineo et al. (2009) [33] reported MCs as the most frequent and abundant cyanotoxins in a study focused on the occurrence and distribution of cyanotoxins from 1989 to 2006 in several Italian lakes, including Lake Bidighinzu. Mariani et al. (2015) [36] found MCs in 71% of the analysed samples (*n* = 62) collected from four Sardinian artificial lakes, including Lake Bidighinzu (75%; *n* = 16), over an 18-month period. During the present study, MCs were detected in all the analysed samples collected from Lake Bidighinzu, but at lower concentrations than in previous studies and never exceeding the World Health Organisation (WHO) guideline of 1 μg L^−1^ MC-LR. MCs showed seasonal dynamics similar to that reported by Mariani et al. (2015) [36], with a maximum in autumn. During MC maxima, Chroococcales dominated (*Cyanocatena*, [33]; *Aphanocapsa*, [36]; *Aphanothece*, *Aphanocapsa* and *Coelosphaerium* in the present study) but not of the *Microcystis* genus, the most involved in MCs CyanoHABs worldwide. Mariani et al. (2015) [36] have already highlighted that species composition during periods of maxima MC concentrations in the studied Sardinian reservoirs differed from those typically reported for other Mediterranean sites, emphasising the importance of long-term studies for the early detection of community changes, especially important when the changes involve harmful species.

The present study reports the first data on BMAA in water samples in Lake Bidighinzu as well as in other Mediterranean aquatic ecosystems, excluding the studies on BMAA bioaccumulation in shellfish from French lagoons [121,122]. Additionally, very few works report seasonal dynamics of BMAA and associated phytoplankton species composition [121,122,123] and its co-occurrence with MCs [124]. Co-occurrence was also assessed in the *M. aeruginosa* and *D. flos-aquae* cell cultures in this study. Interactions of co-occurring BMAA and other cyanotoxins require further investigation [125].

BMAA did not show a clear seasonality in Lake Bidighinzu, peaking in almost all seasons (spring, summer and winter) and coinciding with different cyanobacteria species compositions. This result agreed with BMAA production by numerous cyanobacteria species [27], in addition to other microalgae groups, such as diatoms and dinoflagellates [122,123], and it has a high probability to be present in environments with cyanobacterial growth [27].

## 5. Conclusions

The results from this study encourage further investigation to increase the knowledge of toxic species still debated in the Mediterranean region and on cyanotoxins not investigated enough in this geographical area, as well as worldwide.

The present work has revealed the highest toxicity of *A. pacificum* among the analysed strains and has documented the presence of PSTs also in a Mediterranean strain of *A. tamarense*, highlighting the need for the further investigation of *A. tamarense* strains from different geographical areas in the Mediterranean Sea. The toxin profiles of the strains belonging to the different species considered in this study were clearly differentiated at the species level, confirming the importance of this information. Detailed information on the presence of potentially toxic species or specific strains in a geographic area represents a necessary basis for developing management strategies of the risk [2,126].

This study offers a first estimation of BMAA, both in natural samples and in cyanobacterial cell cultures, in addition to a further documentation of MCs in a Mediterranean eutrophic artificial lake. These BMAA results are of paramount importance, since Sardinian human populations represent a genetic isolate, characterized by a frequency higher than the expected rate of Amyotrophic Lateral Sclerosis and of genetic mutations involved in different neuropathological conditions [127]. Cyanotoxin presence suggests the necessity for specific epidemiologic investigations on related human populations and recommends the broadening of the research to other Sardinian artificial lakes, whose water is used for drinking.

## Figures and Tables

**Table 1:** List of *Alexandrium* isolates considered in this study and reference for toxins, morphology and genetic data.

Species	Strain	Geographic Origin	Year and Source of Culture	Code Site	PSP Toxicity	Reference		
						Toxins	Morphology	Genetic
*A. pacificum*	CNR-ACATS2	Gulf of Olbia, Tyrrhenian	2002, vegetative cell	GO	yes	^†^	^†^	*
*A. pacificum*	CNR-ACATC2	Gulf of Olbia, Tyrrhenian	2002, vegetative cell	GO	yes	^†^	^†^	*
*A. pacificum*	CNR-ACATSRA4	Syracuse harbour, Ionian	2012, vegetative cell	SY	yes	^†^	^†^	**
*A. minutum*	UNISS3	Gulf of Olbia, Tyrrhenian	2012, vegetative cell	GO	yes	^†,§^	^†,§^	^†,§^
*A. minutum*	UNISS4	Gulf of Olbia, Tyrrhenian	2012, vegetative cell	GO	yes	^†,§^	^†,§^	^†,§^
*A. minutum*	CNR-AMIA2PT	Porto Torres harbour, Asinara Gulf	2002, vegetative cell	PT	yes	^†^	^†^	*
*A. minutum*	CNR-AMID6	Syracuse harbour, Ionian	2012, vegetative cell	SY	no	^†^	n.d.	**
*A. minutum*	CNR-AMISY1	Syracuse harbour, Ionian	2012, vegetative cell	SY	no	^†^	n.d.	**
*A. minutum*	CNR-AMIA4	Syracuse harbour, Ionian	2001, vegetative cell	SY	yes	^†^	^#^	^#^
*A. minutum*	CNR-AMIA5	Syracuse harbour, Ionian	2001, vegetative cell	SY	yes	^†^	^#^	^#^
*A. tamarense*	CNR-ATA6PT	Porto Torres harbour, Asinara Gulf	2002, vegetative cell	PT	yes	^†^	^†^	^##^
*A. tamarense*	UNISS5	Gulf of Alghero, Sardinian	2010, resting cyst	GA	no	^†,§^	^†,§^	^†,§^

^†^ This study, ^§^ [46] Stacca, 2013, * [47] Penna et al. 2008, ** [48] Penna et al. 2015, ^#^ [20] Vila et al. 2005, ^##^ [49] Penna et al. 2007, n.d. not described.

**Table 2:** List of *Alexandrium* isolates analysed in this study and relative content in PSTs.

Strain	Code	Site	Toxins (fmol cell^−1^)								
			STX	GTX5	GTX2,3	GTX1,4	C1/2	dcGTX2,3	dcSTX	NeoSTX	Total
*A. pacificum*	CNR-ACATS2	GO	0.229	11.808	0.579	5.129	---	---	---	0.066	17.811
*A. pacificum*	CNR-ACATC2	GO	0.048	4.280	0.622	6.159	---	---	---	0.505	11.614
*A. pacificum*	CNR-ACATSRA4 *	SY	0.008	0.022	0.045	0.057	0.082	<LOD	0.005	<LOD	0.219
*A. minutum*	UNISS3 *	GO	<LOQ	<LOD	0.004	0.013	<LOD	<LOQ	<LOD	<LOD	0.017
*A. minutum*	UNISS4 *	GO	<LOQ	<LOQ	0.001	0.018	<LOD	<LOQ	<LOD	<LOD	0.020
*A. minutum*	CNR-AMIA2PT	GPT	<LOD	<LOD	0.012	0.308	---	---	---	<LOD	0.320
*A. minutum*	CNR-AMID6 *	SY	<LOD	<LOD	<LOD	<LOD	<LOD	<LOD	<LOD	<LOD	<LOD
*A. minutum*	CNR-AMISY1 *	SY	<LOD	<LOD	<LOD	<LOD	<LOD	<LOD	<LOD	<LOD	<LOD
*A. minutum*	CNR-AMIA4	SY	<LOD	<LOD	0.008	0.100	---	---	---	<LOD	0.108
*A. minutum*	CNR-AMIA5	SY	<LOD	<LOD	0.007	0.096	---	---	---	<LOD	0.103
*A. tamarense*	CNR-ATA6PT	GPT	<LOD	0.044	0.007	0.048	---	---	---	<LOD	0.099
*A. tamarense*	UNISS5 *	GA	<LOD	<LOD	<LOD	<LOD	<LOD	<LOD	<LOD	<LOD	<LOD

LOD = limit of detection, LOQ = limit of quantification, --- = toxins not analysed, * concentrated 1:10 times.

**Table 3:** List of cyanobacteria taxa, abundances (cells × 10^6^ L^−1^) and measured cyanotoxins in Lake Bidighinzu in the study period.

Taxa	2014										2015		
	March	April	March	June	July	August	September	October	November	December	January	March	April
*Aphanizomenon flos-aquae*	0.00	0.09	1.32	28.14	68.78	6.98	0.00	0.00	0.00	0.10	0.00	0.00	0.00
*Aphanizomenon* sp.	1.21	0.00	0.00	0.00	0.00	0.00	0.00	0.18	0.08	0.05	0.00	0.00	0.00
*Aphanocapsa incerta*	0.00	0.00	8.92	5.47	0.00	0.00	37.65	223.62	0.54	0.00	0.00	0.00	0.00
*Aphanocapsa* sp.	6.70	0.27	10.86	17.81	2.82	65.24	26.17	30.71	7.41	5.92	10.09	0.10	0.00
*Aphanothece* sp.	0.00	0.00	0.00	0.00	0.34	0.38	0.00	0.00	8.46	4.62	3.02	0.38	0.00
*Chroococcus* sp.	0.00	0.00	0.00	0.00	0.00	0.03	0.02	0.09	0.00	0.00	0.00	0.00	0.00
*Cyanocatena* sp.	0.00	0.00	0.00	0.00	48.12	21.02	308.14	190.01	1.24	0.00	0.00	0.00	0.00
*Coelosphaerium* sp.	0.00	0.00	0.12	0.40	0.92	2.44	1.17	14.90	6.13	2.46	0.59	0.13	0.07
*Dolichospermum flos-aquae*	0.00	0.13	1.58	39.07	0.00	0.00	0.00	0.00	0.00	0.00	0.69	0.00	0.00
*Dolichospermum planctonicum*	0.00	0.00	0.00	0.00	15.05	0.00	0.00	0.00	0.00	0.00	0.00	0.00	0.00
*Dolichospermum* sp.	0.00	0.00	0.15	0.00	0.00	0.00	0.00	0.00	0.00	0.01	0.00	0.05	0.01
*Dolichospermum spiroides*	0.00	0.00	0.00	0.00	0.00	0.00	0.11	0.00	0.00	0.00	0.00	0.00	0.00
*Dolichospermum viguierii*	0.00	0.00	0.00	0.00	0.33	0.00	0.00	0.00	0.00	0.00	0.00	0.00	0.00
*Merismopedia punctata*	0.00	0.00	0.00	0.63	0.00	0.00	0.00	0.09	0.94	1.08	0.00	0.00	0.10
*Merismopedia tenuissima*	0.05	0.00	0.50	0.93	0.13	1.22	43.10	13.20	4.68	2.01	0.04	0.00	0.00
*Microcystis aeruginosa*	0.00	0.00	0.00	0.00	0.00	0.00	3.26	0.99	0.00	0.12	0.15	0.00	0.00
*Microcystis* sp.	0.00	0.00	0.00	0.00	0.00	0.07	1.74	0.70	0.29	0.00	0.00	0.00	0.00
*Planktolyngbya* sp.	0.00	0.00	0.00	0.00	0.00	0.00	0.00	0.00	0.00	0.00	0.00	3.92	0.00
*Planktothrix* sp.	0.00	0.00	0.00	0.00	0.00	0.00	0.00	0.00	0.00	0.00	0.41	0.00	0.00
*Pseudanabaena* sp.	0.12	0.00	0.00	0.00	0.06	0.00	0.03	0.14	0.00	0.00	0.44	0.20	0.62
Pseudanabaenaceae	0.00	0.00	0.00	0.00	0.00	0.00	0.00	0.00	4.61	7.81	0.26	0.09	0.00
*Snowella lacustris*	15.42	10.64	7.62	0.00	0.00	0.04	0.20	0.00	0.00	0.22	11.67	1.32	7.09
*Snowella* sp.	0.00	0.00	0.00	0.73	0.00	0.00	0.00	0.00	0.23	0.24	0.00	0.00	0.00
*Woronichinia compacta*	0.00	0.00	0.00	0.00	0.00	0.00	0.00	0.00	0.00	0.00	0.42	0.29	0.00
*Woronichinia naegeliana*	0.00	0.18	0.00	0.00	0.00	0.00	0.00	0.00	0.00	0.04	0.00	0.12	0.00
*Woronichinia* sp.	0.00	0.00	0.00	0.00	0.00	0.00	0.00	0.00	0.00	0.00	0.01	0.07	0.00
Other Cyanophyceae	0.00	0.00	1.39	0.04	0.00	0.00	0.00	0.00	0.00	0.00	0.00	0.00	0.00
Total abundance (cells × 10^6^ L^−1^)	23.5	11.3	32.5	93.2	136.6	97.4	421.6	474.6	34.6	24.7	27.8	6.7	7.9
MCs (µg L^−1^)	0.09	0.11	0.05	0.31	0.25	0.30	0.38	0.59	0.95	0.67	0.53	0.42	0.21
BMAA (µg L^−1^)	5.87	1.43	2.93	17.84	5.90	2.96	14.78	17.67	5.44	1.61	9.00	10.23	10.15

## Supplementary Materials

The following are available online at www.mdpi.com/2076-2607/5/4/72/s1, Table S1: List of sequences used in the phylogenetic trees for the ITS-5.8S rDNA regions reporting species names, strains or isolates, sampling locations, clades, and GenBank accesion numbers. In bold the sequences obtained in the present study, Table S2: List of sequences used in the phylogenetic trees for the LSU rDNA region reporting species names, strains or isolates, sampling locations, clades, and GenBank accesion numbers. In bold the sequences obtained in the present study.
