# Supplementary material for: Paralytic Shellfish Toxins and Cyanotoxins in the Mediterranean: New Data from Sardinia and Sicily (Italy)
Source: Microorganisms. 2017 Nov 16;5(4):72. doi: 10.3390/microorganisms5040072 (PMC5748581; doi:10.3390/microorganisms5040072)
Supplement: Supplementary file 1 [file microorganisms-05-00072-s001.pdf]

Table S1 List of sequences used in the phylogenetic trees for the ITS-5.8S rDNA regions reporting species names, strains or isolates, sampling locations, clades, and GenBank accesion numbers. In bold the sequences obtained in the present study.

Species	Strain	Sampling Location	Clade	Accession Number
A. affine	IEO-PA4V	Spain: Ria de Vigo, Galicia		AJ632094
A. affine	IEO-PA8V	Spain: La Linea, Gibraltar		AJ632095
A. affine	H1	Japan: Harima Nada		AB006995
A. lusitanicum	GT-PORT	Portugal: Lagoa de Obidos	Global clade	EU707539
A. lusitanicum	AL2V	Spain: Ria de Vigo, Galicia	Global clade	EU707499
A. minutum	**UNISS3**	**Italy: Olbia, Tyrrhenian Sea, Mediterranean Sea, Sardinia**	**Global clade**	**MF540537**
A. minutum	**UNISS4**	**Italy: Olbia, Tyrrhenian Sea, Mediterranean Sea, Sardinia**	**Global clade**	**MF540538**
A. minutum	CBA28	Italy: Olbia, Tyrrhenian Sea, Mediterranean Sea, Sardinia	Global clade	FR668134
A. minutum	CNR-AMIA4OL	Italy: Olbia, Tyrrhenian Sea, Mediterranean Sea, Sardinia	Global clade	AJ514921
A. minutum	SAAM12	South Africa: Cape Town	Global clade	EU707547
A. minutum	IEO-AL8C	Spain: Arenys, Catalan Sea, Mediterranean Sea	Global clade	AJ532914
A. minutum	SZN030	Italy: Gulf of Naples, Tyrrhenian Sea, Mediterranean Sea	Global clade	EU707549
A. minutum	AMFL	England: Fleet Lagoon	Global clade	EU707521
A. minutum	AM1	South Africa: Cape Town	Global clade	EU707511
A. minutum	AL5T	Italy: Gulf of Trieste, Adriatic Sea, Mediterranean Sea	Global clade	EU707505
A. minutum	AMIR-1	Ireland: Cork Harbor	Global clade	EU707523
A. minutum	CNR-AMIA1	Italy: Siracusa, Ionian Sea, Mediterranean Sea, Sicilia	Global clade	AJ621734
A. minutum	CNR-AMIA4PT	Italy: Porto Torres, Mediterranean Sea, Sardinia	Global clade	AJ514920
A. minutum	CNR-AMIA2OL	Italy: Olbia, Tyrrhenian Sea, Mediterranean Sea, Sardinia	Global clade	AJ532908
A. minutum	CNR-AMIA2PT	Italy: Porto Torres, Mediterranean Sea, Sardinia	Global clade	AJ532909
A. minutum	VGO663	Italy: Olbia, Tyrrhenian Sea, Mediterranean Sea, Sardinia	Global clade	AM238453
A. minutum	LAC27	Italy: Gulf of Trieste, Adriatic Sea, Mediterranean Sea	Global clade	AJ005050
A. catenella	ACC01	Chile	Group I, (NA)	AJ272120
A. fundyense	GtCA29A	USA: Cape Ann, North Atlantic	Group I, (NA)	NR
A. tamarense	04-197-A1	United Kingdom: Scotland, Stonehaven	Group I, (NA)	FJ042687
A. tamarense	04-197-30	United Kingdom: Scotland, Stonehaven	Group I, (NA)	FJ042686
A. tamarense	FK-788A/B	Japan: Funka Bay	Group I, (NA)	AB006994; AB006993
A. tamarense	MDQ1096	Argentina: Mar de la Plata, West Pacific	Group I, (NA)	AM292306
A. tamarense	PW06	USA: Port Benny, North Pacific	Group I, (NA)	NR
A. tamarense	OK875-1A/B	Japan: Okirai Bay, East Pacific	Group I, (NA)	NR
A. tamarense	AT4A/B	Japan: Harima Nada, East Pacific	Group I, (NA)	NR
A. tamarense	OFX191-1A/B	Japan: Ofunato Bay, East Pacific	Group I, (NA)	NR
A. tamarense	At503A-A/B	Japan: Mikawa Bay, East Pacific	Group I, (NA)	NR
A .tamarense	OFX151A	Japan: Ofunato Bay, East Pacific	Group I, (NA)	NR
A. tamarense	CNR-ATAA1	Italy: Taranto, Ionian Sea, Mediterranean Sea, Puglia	Group II, (ME)	AJ491152
A. tamarense	CNR-ATAA3	Italy: Taranto, Ionian Sea, Mediterranean Sea, Puglia	Group II, (ME)	AM292308
A. tamarense	IEO-VGO654	Spain: Maiorca, Balearic Sea coast	Group II, (ME)	AM238650
A. tamarense	IEO-VGO553	Greece: Kavala, Aegean Sea coast	Group II, (ME)	AM238651
A. tamarense	SZN-01	Italy: Gulf of Naples, Tyrrhenian Sea, Mediterranean Sea	Group II, (ME)	AM238652
A. tamarense	CNR-OR3	Italy: Oristano, Mediterranean Sea, Sardinia	Group II, (ME)	AM292307

A. fundyense	CCMP1719	USA	Group III, (WE)	DQ444290
A. tamarense	CCMP115	United Kingdom: England, Plymouth	Group III, (WE)	JF521640
A. tamarense	S06-010-01	United Kingdom: Scotland, Weisdale, Shetland	Group III, (WE)	FJ042685
A. tamarense	TL-A3200907F5	France: Thau Lagoon, Mediterranean Sea	Group III, (WE)	FR686537
A. tamarense	CNR-ATA4PT	Italy: Porto Torres, Mediterranean Sea, Sardinia	Group III, (WE)	AJ514906
A. tamarense	CNR-ATA6PT	Italy: Porto Torres, Mediterranean Sea, Sardinia	Group III, (WE)	AJ514907
A. tamarense	CCMP 116	Spain: Ria de Vigo, Galicia	Group III, (WE)	AJ005047
A. tamarense	WKS-1	Japan: Kushimoto, East Pacific	Group III, (WE)	AB006991
A. tamarense	**UNISS5**	**Italy: Gulf of Alghero, Mediterranean Sea, Sardinia**	**Group III, (WE)**	**MF540539**
A. tamarense	AT-6	China	Group III, (WE)	DQ176666
A. catenella	OFX102	Japan: Ofunato Bay, East Pacific	Group IV, (TA)	NR
A. catenella	OFY101	Japan: Ofunato Bay, East Pacific	Group IV, (TA)	NR
A. catenella	TNY11	Japan: Tanabe Bay, East Pacific	Group IV, (TA)	NR
A. catenella	TNX22	Japan: Tanabe Bay, East Pacific	Group IV, (TA)	NR
A. catenella	Y-2	Japan: Yamakawa, East Pacific	Group IV, (TA)	NR
A. catenella	K0-3	Japan: Uranouchi Inlet,East Pacific	Group IV, (TA)	NR
A. catenella	IEO-709	Spain: Tarragona, Catalan Sea, Mediterranean Sea	Group IV, (TA)	AJ968683
A. catenella	IEO-715	Spain: Tarragona, Catalan Sea, Mediterranean Sea	Group IV, (TA)	AJ968681
A. catenella	IEO-816	France: Thau Lagoon, Mediterranean Sea	Group IV, (TA)	AJ968680
A. catenella	IEO-AC2C	Spain: Barcelona, Catalan Sea, Mediterranean Sea	Group IV, (TA)	AJ532912.
A. catenella	CNR-ACATA4	Italy: Olbia, Tyrrhenian Sea, Mediterranean Sea, Sardinia	Group IV, (TA)	AJ532910
A. catenella	ATTL02	France: Thau Lagoon, Mediterranean Sea	Group IV, (TA)	AJ608264
A. catenella	ATTL01	France: Thau Lagoon, Mediterranean Sea	Group IV, (TA)	AJ608263
A. catenella	CSIC-5T	Spain: Tarragona, Catalan Sea, Mediterranean Sea	Group IV, (TA)	AJ580325
A. catenella	CNR-ACATS3	Italy: Olbia, Tyrrhenian Sea, Mediterranean Sea, Sardinia	Group IV, (TA)	AJ580320
A. catenella	CNR-ACATS1	Italy: Olbia, Tyrrhenian Sea, Mediterranean Sea, Sardinia	Group IV, (TA)	AJ580319
A. catenella	CNR-ACATS2	Italy: Olbia, Tyrrhenian Sea, Mediterranean Sea, Sardinia	Group IV, (TA)	AJ580318
A. catenella	CNR-ACATC2	Italy: Olbia, Tyrrhenian Sea, Mediterranean Sea, Sardinia	Group IV, (TA)	AJ580317
A. catenella	ACTEM17	France: Thau Lagoon, Mediterranean Sea	Group IV, (TA)	FM211470
A. catenella	AC0409-13	Japan: Akasaki Seaport	Group IV, (TA)	FM211466
A. catenella	AC0310-06	Japan: Kita Nada, Kagawa	Group IV, (TA)	FM211462
A. catenella	AC0206-18	Japan: Ago Bay, Mie	Group IV, (TA)	FM211460
A. catenella	AC0202-15	Japan: Uchino-Umi, Tokushima	Group IV, (TA)	FM211457
A. catenella	ACDH	China	Group IV, (TA)	DQ176658
A. catenella	MI7	Japan: Harima Nada	Group IV, (TA)	AB006990
A. catenella	TL-C3111007G3	France: Thau Lagoon, Mediterranean Sea	Group IV, (TA)	FR686536
A. tamarense	AT5-1	China	Group IV, (TA)	DQ176662
A. catenella	UNISS6	Italy: Santa Giusta Lagoon, Mediterranean Sea, Sardinia	Group IV, (TA)	MF574310
G. spinifera	CCMP 409	USA: West Boothbay Harbor, MA		EU532487

Note: The following acronyms, abbreviations and symbols indicate respectively:
NA = North American clade, ME = Mediterranean clade, WE = Western European clade, TA = Temperate Asian clade
NR = GenBank sequence not reported

Table S2 List of sequences used in the phylogenetic trees for the LSU rDNA region reporting species names, strains or isolates, sampling locations, clades, and GenBank accesion numbers. In bold the sequences obtained in the present study.

Species	Strain	Sampling Location	Clade	Accession Number
A. affine	AABCV-1	Mexico: Gulf of California, Notrh Pacific Ocean		AY152706
A. affine	PA5V	Spain: Ria de Vigo, Galicia		NR
A. affine	CU-1	Thailand: Pacific Ocean		U44935
A. lusitanicum	18-1T	Portugal: Lagoa de Obidos	Global clade	EU707456
A. lusitanicum	18-1NT	Portugal: Lagoa de Obidos	Global clade	EU707455
A. lusitanicum	AL2V	Spain: Ria de Vigo, Galicia	Global clade	EU707460, AY962837
A. lusitanicum	AL-1	Portugal	Global clade	JF906999
A. minutum	**UNISS3**	**Italy: Olbia, Tyrrhenian Sea, Mediterranean Sea, Sardinia**	**Global clade**	**MF540550**
A. minutum	**UNISS4**	**Italy: Olbia, Tyrrhenian Sea, Mediterranean Sea, Sardinia**	**Global clade**	**MF540551**
A. minutum	AMAD16	Australia: Adelaide	Global clade	JF521633
A. minutum	AMIR-1	Ireland: Cork Harbor	Global clade	EU707473, AY962848
A. minutum	AMFL	England: Fleet Lagoon	Global clade	EU707471
A. minutum	AM1	France: Morlaix Bay	Global clade	EU707466 ; AY962843
A. minutum	AMAD06	Australia: Port River	Global clade	U44936
A. minutum	CCMP113	Spain: Ria de Vigo, Galicia	Global clade	JF521634
A. minutum	LAC27	Italy: Gulf of Trieste, Adriatic Sea, Mediterranean Sea	Global clade	AY962842
A.minutum	AMNZ02	New Zeland: Anakoha Bay	Pacific Clade	EU707477
A.minutum	AMNZ01	New Zeland: Croisilles Harbor	Pacific Clade	EU707476
A.minutum	AMBOPO06	New Zeland: Tauranga Harbor	Pacific Clade	EU707468,AY962846
A.minutum	AMBOPO14	New Zeland	Pacific Clade	EU707469,AY962847
A. catenella	ACC01	Chile	Group I, (NA)	AY268597
A. fundyense	GtCA29	USA: Cape Ann, North Atlantic	Group I, (NA)	NR
A. tamarense	PW06	USA: Port Benny, North Pacific	Group I, (NA)	U44927
A. tamarense	04-197-A1	Scotland	Group I, (NA)	FJ042682
A. tamarense	04-197-30	Scotland	Group I, (NA)	FJ042681
A. tamarense	Alex61-1	Scotland: Firth of Forth	Group I, (NA)	AJ303445
A. tamarense	Alex61-2	Scotland: Firth of Forth	Group I, (NA)	AJ303446
A. tamarense	SZN01	Italy: Gulf of Naples, Tyrrhenian Sea, Mediterranean Sea	Group II, (ME)	AJ535368
A. tamarense	SZN08	Italy: Gulf of Naples, Tyrrhenian Sea, Mediterranean Sea	Group II, (ME)	AJ535369
A. tamarense	SZN19	Italy: Gulf of Naples, Tyrrhenian Sea, Mediterranean Sea	Group II, (ME)	AJ535370
A. tamarense	SZN21	Italy: Gulf of Naples, Tyrrhenian Sea, Mediterranean Sea	Group II, (ME)	AJ535374
A. tamarense	CCMP115	England: Plymouth	Group III, (WE)	JF521640

A. tamarense	Pgt183	England: Plymouth	Group III, (WE)	U44930
A. tamarense	S06-010-01	Scotland: Shetland, Weisdale	Group III, (WE)	FJ042673
A. tamarense	Alex31.6	Ireland: Cork Harbour	Group III, (WE)	AJ303433
A. tamarense	WKS-1	Japan: Kushimoto, East Pacific	Group III, (WE)	NR
A. tamarense	S07-035-01	Scotland: Laxfirth, Shetland	Group III, (WE)	FJ042680
A. tamarense	Ply 173	England: Plymouth	Group III, (WE)	AJ308587
A. tamarense	TL-A3200907F5	France: Thau Lagoon	Group III, (WE)	FR686537
A. tamarense	**UNISS5**	**Italy: Gulf of Alghero, Mediterranean Sea, Sardinia**	**Group III, (WE)**	**MF540552**
A. catenella	DPC95b	South Korea: Dadaepo	Group IV, (TA)	AY082051
A. catenella	OF101	North Japan: Ofunato Bay	Group IV, (TA)	U44931
A. catenella	ACT1	France: Thau Lagoon, Mediterranean Sea	Group IV, (TA)	FM211468
A.catenella	ATT98	France: Thau Lagoon, Mediterranean Sea	Group IV, (TA)	AF318220
A.catenella	ACTRA02	Australia: Triabunna, Tasmania	Group IV, (TA)	AY338754
A. catenella	AC0206-18	Japan: Ago Bay, Mie	Group IV, (TA)	FM211460
A. catenella	AC0409-13	Japan: Akasaki Seaport	Group IV, (TA)	FM211466
A. catenella	AC0409-08	Japan: Akasaki Seaport	Group IV, (TA)	FM211465
A. catenella	AC0310-06	Japan: Kita Nada, Kagawa	Group IV, (TA)	FM211462
A.catenella	AC0310-01	Japan: Kita Nada, Kagawa	Group IV, (TA)	FM211461
A. catenella	ACTEM17	France: Thau Lagoon, Mediterranean Sea	Group IV, (TA)	FM211470
A. catenella	ACTEM9	France: Thau Lagoon, Mediterranean Sea	Group IV, (TA)	FM211469
A. catenella	AC0202-15	Japan: Uchino-Umi, Tokushima	Group IV, (TA)	FM211457
A. catenella	TL-C3111007G3	France: Thau Lagoon, Mediterranean Sea	Group IV, (TA)	FR686536
A. catenella	UNISS6	Italy: Oristano, Mediterranean Sea, Sardinia	Group IV, (TA)	MF574309
A. catenella	LAC35	Australia: Port Phillip Bay	Group IV, (TA)	AY268610
A. tamarense	ATCI01-1	China: Dai Ya Bay	Group IV, (TA)	AY268612
A. tamarense	AT5-3	South China	Group IV, (TA)	JF906993
A. tamarense	ATBB01	Australia: Bell Bay, Tasmania	Group V, (TASM)	U44933
G. spinifera	GSA0602	Italy: Cesenatico, Adriatic Sea, Mediterranean Sea		EU805591

Note: The following acronyms, abbreviations and symbols indicate respectively:
NA = North American clade, ME = Mediterranean clade, WE = Western European clade, TA = Temperate Asian clade, TASM = Tasmanian clade
NR = GenBank sequence not reported
